# A natural antipredation experiment: predator control and reduced sea ice increases colony size in a long-lived duck

**DOI:** 10.1002/ece3.735

**Published:** 2013-09-01

**Authors:** Sveinn A Hanssen, Børge Moe, Bård-Jørgen Bårdsen, Frank Hanssen, Geir W Gabrielsen

**Affiliations:** 1 Arctic Ecology Department, Norwegian Institute for Nature Research, Fram CentreN-9296, Tromsø, Norway; 2 Unit for Terrestrial Ecology, Norwegian Institute for Nature ResearchNO-7485, Trondheim, Norway; 3 Norwegian Polar Institute, Fram CentreN-9296, Tromsø, Norway

**Keywords:** Carrying capacity, climate change, high Arctic, population growth rate, predator effects, sea ice, Svalbard

## Abstract

Anthropogenic impact on the environment and wildlife are multifaceted and far-reaching. On a smaller scale, controlling for predators has been increasing the yield from local natural prey resources. Globally, human-induced global warming is expected to impose severe negative effects on ecosystems, an effect that is expected to be even more pronounced in the scarcely populated northern latitudes. The clearest indication of a changing Arctic climate is an increase in both air and ocean temperatures leading to reduced sea ice distribution. Population viability is for long-lived species dependent on adult survival and recruitment. Predation is the main mortality cause in many bird populations, and egg predation is considered the main cause of reproductive failure in many birds. To assess the effect of predation and climate, we compared population time series from a natural experiment where a trapper/down collector has been licensed to actively protect breeding common eiders *Somateria mollissima* (a large seaduck) by shooting/chasing egg predators, with time series from another eider colony located within a nature reserve with no manipulation of egg predators. We found that actively limiting predator activity led to an increase in the population growth rate and carrying capacity with a factor of 3–4 compared to that found in the control population. We also found that population numbers were higher in years with reduced concentration of spring sea ice. We conclude that there was a large positive impact of human limitation of egg predators, and that this lead to higher population growth rate and a large increase in size of the breeding colony. We also report a positive effect of warming climate in the high arctic as reduced sea-ice concentrations was associated with higher numbers of breeding birds.

## Introduction

Throughout history humans have been shaping their environment, and anthropogenic impact on the environment and wildlife has been accelerating in line with increasing industrialization and human global population growth. On a smaller scale, humans have affected wildlife by, for example, hunting and in some areas humans have increased their hunting yield by protecting the preferred prey from other predators, an activity that has persisted to present times (e.g., Campbell 1998). In more recent times and on a more global scale, human-induced global warming is moving consequences of human activity from local impact associated with densely populated areas, to even the remotest corners of the world; climate change is worldwide and it is even more pronounced in the scarcely populated northern latitudes (e.g., Serreze et al. 2000; Tebaldi et al. 2006; Benestad 2007).

Population viability for long-lived species generally depends on two parameters: adult survival and recruitment (e.g., Stearns 1992), and for many birds predation is the main cause of mortality (Newton 1998). During the breeding season, egg predation is the main cause of reproductive failure in many birds (O'Connor 1991; Martin 1993). The breeding season is thus a vulnerable period were several challenges may affect offspring production and thereby population viability. Recent studies have shown that birds may avoid areas where they have previously experienced egg predation (Hanssen and Erikstad 2013), or areas where they experience signs of predators (Forsman et al. 2013). To assess the effect of predation, experiments have been performed where predators have been decimated or removed (review by Côté and Sutherland 1997). Côté and Sutherland (1997) found that effects of predator removal on hatching success and postbreeding population sizes were quite consistent and large, while the effects on breeding population sizes were not as pronounced. In order to explain this, more studies on the potential effect of predators on breeding population sizes are necessary (Côté and Sutherland 1997).

The clearest indication of a changing Arctic climate is an increase in both air and ocean temperatures. The latest predictions of the change varies, but it seems reasonable to assume a 2.5°C rise in mean air temperature by 2050, and a further increase as high as 5°C by the end of the century (ACIA 2005; Benestad 2011). This warming of the Arctic is already leading to a decline in Arctic sea ice shown through a decrease in sea ice extent, reduced ice thickness, and lower ice age, with more first-year ice (Rothrock et al. 1999; Parkinson and Cavalieri 2002; Nghiem et al. 2007; Comiso et al. 2008; Walsh 2008; Kwok and Rothrock 2009). Climate change has the potential for altering population dynamics of wildlife (Post and Stenseth 1999), but the direction of this impact may be difficult to predict (e.g., Bårdsen et al. 2011). Some studies have documented negative effects of climate change on bird population dynamics (Barbraud and Weimerskirch 2001; Jenouvrier et al. 2003; Both et al. 2006), whereas some have documented positive effects (Kitaysky and Golubova 2000; Gaston et al. 2005a; Halupka et al. 2008). It has been suggested that global warming should benefit organisms living at the northern limit of its species distribution (Sparks et al. 2002; Gaston et al. 2005b; but see Sandvik et al. 2008). Global warming may lead to changes in population levels and distribution for many wildlife species, through shifting phenology where the timing of key life-history elements may be disrupted (Easterling et al. 2000; Stenseth and Mysterud 2002; Johnston et al. 2005; Gaston et al. 2009). For instance, climatic conditions prebreeding and during breeding may affect the number of offspring produced, either directly or indirectly by affecting food resources (e.g., Sæther et al. 2004). There is increasing evidence that changes are already taking place in Arctic food webs (Ellingsen et al. 2008) leading to changes in the diet and reproductive performance of higher predators such as seals and seabirds (Bluhm and Gradinger 2008; Kovacs and Lydersen 2008; Laidre et al. 2008; Moe et al. 2009).

**Figure 1:**
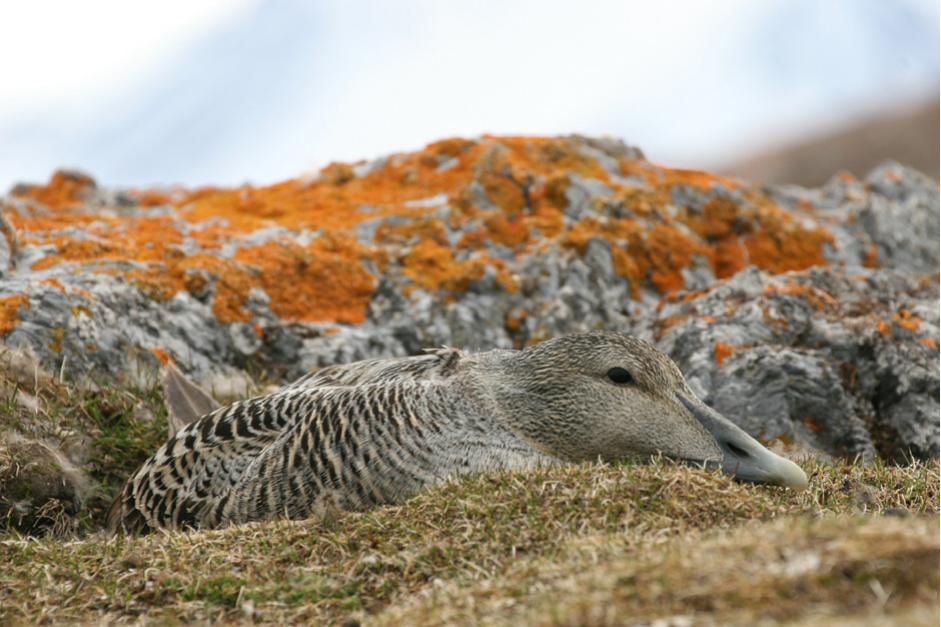
Female common eider *Somateria mollissima* incubating her eggs in Kongsfjorden, Spitsbergen Svalbard. Photo: Børge Moe.

The common eider (Fig. 1) is a long-lived sea-duck that in Europe breeds from the Netherlands in the south with Svalbard and Franz Josef Land in the High Arctic as the northern distribution limit. Some eider populations (including the High Arctic populations) migrate south during winter, indicating that the High Arctic climate is too harsh to sustain these birds during winter. The birds preferably breed on islets to avoid terrestrial nest predators and they are unwilling to start breeding until the sea ice around the islets is melted (Mehlum 1991a; Svendsen et al. 2002; Chaulk and Mahoney 2012). Eider ducks in the Arctic suffer from high levels of nest predation from a range of species (Ahlén and Andersson 1970; Mehlum 1991b; Noel et al. 2005). In Iceland, the birds are important to the Icelandic duck down industry and breeding eider hens are actively protected against predators (Doughty 1979; Skarphédinsson 1996). Also in Svalbard, some trappers still collect eider down, and one breeding colony has been actively managed as an eider farm with predator control by killing or chasing away egg predators such as Arctic fox (*Vulpes lagopus*), Polar bear (*Ursus maritimus*), Arctic skua (*Stercorarius parasiticus*), and Glaucous gull (*Larus hyperboreus*).

**Figure 2:**
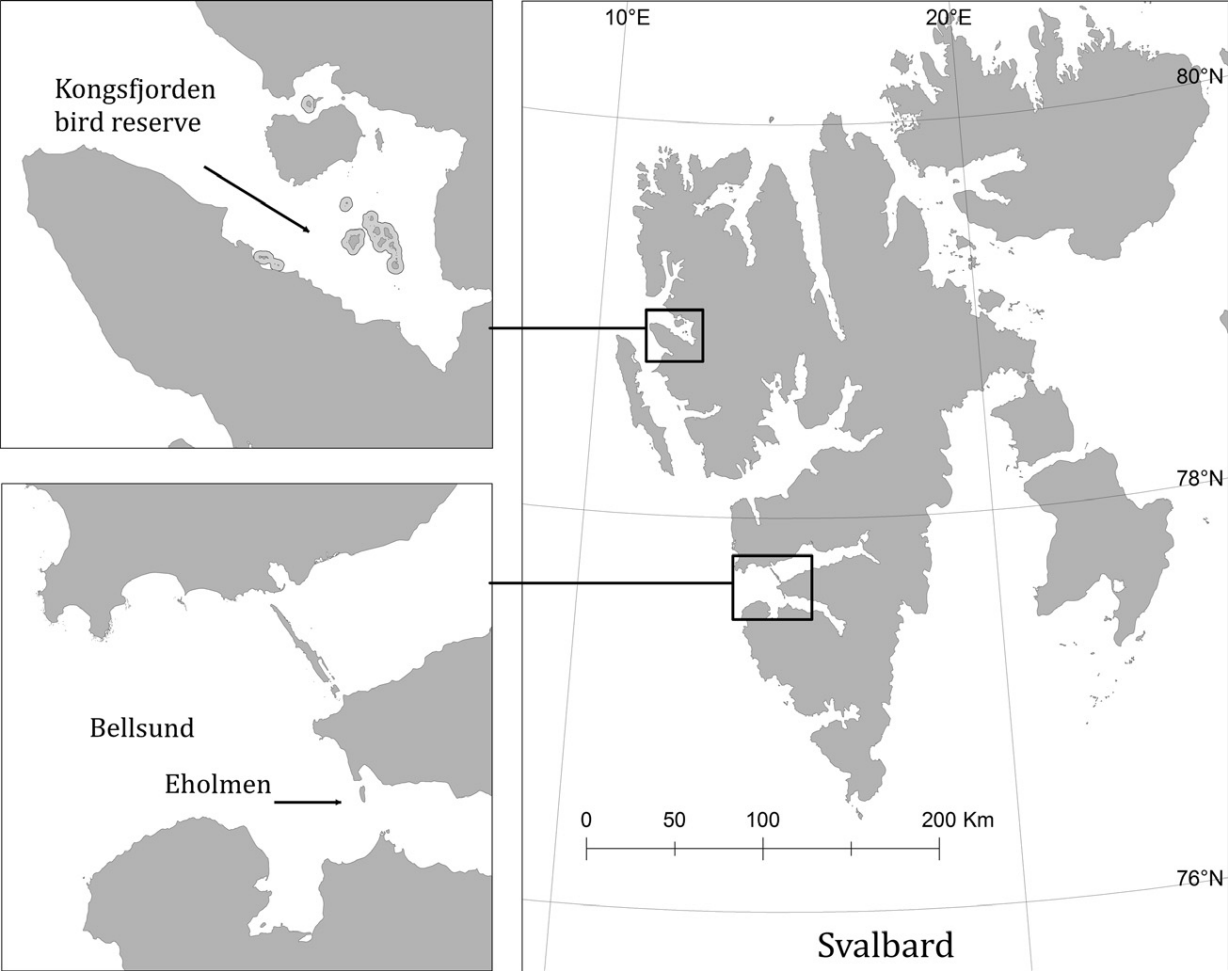
Map of the Svalbard Archipelago with the two study colonies Eholmen (predator removal population) and Kongsfjorden (control population), located at the western coast of the island Spitsbergen.

In this study, we aim to assess the effect of predation and climate on breeding population sizes of high-arctic common eiders from Svalbard. This is made possible by comparing population time series containing 23 and 30 years of population data from two different colonies (Fig. 2). In one colony (Ehomen) a trapper/down collector has been licensed to actively protect the breeding eiders against egg predation by shooting/chasing egg predators, whereas the other colony (Kongsfjorden) is located within a bird reserve with no manipulation of egg predators. To assess the effect of climate on the predator removal- and control colony, we analyzed population sizes in relation to a suit of climatic parameters. We included the current years mean April ice concentration and temperature to assess the potential impact of local climate during spring, and the North Atlantic Oscillation winter index (NAO_W_) to evaluate the potential effect of large-scale winter climate. We also included summer (July) temperature with a 2-year lag in order to see if summer temperature at hatching had an impact on recruitment rate since eider hens may start breeding when they are 2-year old (range 2–5 years) (e.g., Hario and Rintala 2009; Descamps et al. 2011: Table 2). See for example, Bårdsen and Tveraa (2012:365) for a discussion on the use of large-scale climatic indices versus local climatic measures.

## Material and Methods

### Study protocol

Both studied eider colonies are located at West Spitsbergen, Svalbard. The control population is located in Kongsfjorden (Lovénøyane: 78°55'55″N 12°17'33″E) where counts were performed annually from 1981 to 2011 (except for 1988, 1992, and 1994). The experimental population, that is, where predation limitation occurred, is located on Eholmen (Bellsund: 77°35'50″N 14°54'50″E), and counts were performed annually in the population from 1987 to 2010 (except for 2005). The distance between the two colonies is ∼160 km. The Kongsfjorden population (hereafter “control population”) numbers consist of data from 14 islets with a total area of 1.31 km^2^, whereas Eholmen (hereafter “predator removal population”) is on one islet of 0.49 km^2^. The islands in the control population vary in size from 0.01 to 30 ha, and they range from low islets to the larger islands that reach an elevation of up to 35 m asl. The islands contain larger areas with sparse tundra vegetation, rarely exceeding 5 cm in height (Mehlum 2012). The predator removal population nests on an island with similar vegetation as the control population with a maximum elevation of 36 m asl. The eider nests can be found scattered in all parts of the islands, both the vegetated parts and on gravel beaches (Mehlum 2012).

Breeding usually starts late May to early June, possibly related to local ice conditions (Mehlum 1991a). The nest counts were performed after most hens had started incubating, and before the first nests started to hatch (Mehlum 1991a). In the control population, each breeding islet was counted once each year. The predator removal population was counted several times each season, and nest tags were used to separate new nests from older ones. In order to protect Eholmen from egg-eating predators the trapper/down collector lived in the middle of the eider colony during the whole breeding period every year. Egg-eating predators like Arctic fox, Arctic skua, and Glaucous gull were chased away or, in some instances shot when attempting to raid the colony. When Polar bears relatively infrequently visited the colony, they were chased away or they left when becoming aware of the human presence.

### Data

*Predation manipulation*. A factor variable where each manipulation group (control [Kongsfjorden] and predator removal [Eholmen]) acted as levels.

*Population density* [*D*_*t*_ = log_e_(*N* km^−2^)]. A continuous variable denoting the population density based on the total number of nests observed divided by the size of the study area for each population. We transformed this variable using the natural logarithm. Four missing values were replaced with imputed values based on predictions from a model in which population density was modeled as a function of time and manipulation (Data S1).

*Lagged population density* (1 year lag; *D*_*t*−1_). A continuous variable denoting population density with a lag of 1 year (i.e., at *t*−1).

*Population growth rate* [*λ* = log_e_(*D*_*t*+1_/*D*_*t*_)]. A continuous variable denoting population growth from 1 year (*t*) to the next (*t* + 1). This variable is interpreted as follows: (1) *λ* = 0 means no change in population density from *t* to *t* + 1 (i.e., *D*_*t*+1_ = *D*_*t*_); (2) *λ* < 0 means that *D*_*t*+1_ < *D*_*t*_; and (3) *λ* > 0 means that *D*_*t*+1_ > *D*_*t*_.

*Year* (year). A continuous variable denoting time.

*Lagged July temperature* (2 year lag; °C). A continuous variable denoting the detrended average July temperature for Longyearbyen Airport 2 years back in time (i.e., at *t*−3: Data S2).

*April temperature* (°C). A continuous variable denoting the detrended average April temperature for Longyearbyen Airport (Data S2). Temperatures were downloaded from http://www.eklima.no.

*North Atlantic Oscillation winter index (NAO*_*W*_*; relative*). A continuous variable denoting the detrended station based winter NAO for each year (December–March; Data S2). The station based NAO indices were downloaded from https://climatedataguide.ucar.edu/sites/default/files/cas_data_files/asphilli/nao_station_djfm_0.txt.

*Ice concentration (%)*. A continuous variable denoting the detrended ice concentration for April each year (Data S2). Ice concentration data were obtained from the Norwegian Meteorological Institute, using the average ice concentration across two 1° boxes (bounded by 78–79°N 10–11°E and 76–77°N 15–16°E) as an index for West Spitsbergen (for more details, see Moe et al. 2009).

*Intrinsic rate of increase (r)*. The theoretic growth when density is zero, calculated for each colony by the Ricker model (see below).

*Carrying capacity (K)*. The density that corresponds to zero population growth, calculated for each colony by the Ricker model (see below).

### Statistical analyses

Statistical analyses and plotting of results were carried out in *R* (R Development Core Team 2011). All tests were two-tailed, and the null-hypothesis was rejected at an α-level of 0.05. We used the treatment contrast comparing predator removal to the control population which is the baseline level, and Wald statistics to test if estimated parameters were significantly different from zero. The underlying assumption, that is, the residual distribution, behind the models was assessed using the built-in plotting diagnostics tool for the different libraries used. Roughly, we performed three different analyses, where the specific aim in each analysis was to estimate the effect of predation removal (i.e., comparing control vs. predator removal) for:

1. temporal trends in population density;
2. population dynamics, where we assessed both the intrinsic growth rate (*r*) and density-dependent regulation, that is, the carrying capacity (*K*), using the Ricker model; and
3. the effect of key climatic predictors.

#### Analysis 1 – temporal trends in population density

We fitted generalized additive models (GAM) to the data using the gam function in the mgcv library (Wood 2012) in *R* where we used thin plate regression splines to model potential nonlinear effects of continuous variables (possible also in interaction between the two colonies). We used a gamma (*γ*) of 1.4 in order to increase the cost to each effective degree of freedom to avoid overfitting (Wood 2006). Population density, that is, log_e_(*D*_*t*_), was used as the response, whereas the interaction between colonies and year (i.e., temporal trends was modeled separately by area) were used as predictors. Technically, the models were fitted using a log-link function and a Gaussian distribution. One of the advantages of GAM is that the degree of complexity or smoothness, represented by the effective degrees of freedom (edf), within the limits set by “*k*”, which were set to 4 in this study, can be selected objectively (Wood 2006). The edf takes values between 0 and ∞ where higher edf values produce more nonlinear smoothing (Zuur et al. 2009). Plotting of results with respect to each predictor was performed keeping all the other predictors at their average values.

#### Analysis 2 – population dynamics: the Ricker model

We fitted the Ricker model to the time series data for the two colonies separately in order to estimate the two parameters of interest in the Ricker equation (which is a model that predicts λ as function of population abundance; see e.g., Morris and Doak 2002): (1) the intrinsic rate of increase (*r*), which is the theoretic growth when density is zero, and (2) the carrying capacity (*K*), which is the density that corresponds to zero population growth. In this analysis, we used log_e_(λ), where λ = *D*_*t*+1_/*D*_*t*_, as the response and population density (*D*_*t*_) using the nls function in the library nlme (Pinheiro et al. 2012) in R (see also Pinheiro and Bates 2000; Zuur et al. 2009). We tried to fit the Theta-logistic model (Morris and Doak 2002) to our data, but the added complexity resulted in convergence issues for the data from the predator removal colony. In the control colony, however, the Ricker model was selected over the Theta-logistic model (results not shown). Consequently, we fitted the Ricker model, which is actually a special case of the more general Theta-logistic model, to data from both colonies and compared the estimated parameters across areas.

#### Analysis 3 – climatic effects

In this analysis, we fitted models using population density [i.e., log_e_(*D*_*t*_)] as the response, and a set of different climatic measures that we had a priori expectations to as predictors. We were particularly interested in the effects of the following key predictors: (1) Lagged July temperature, (2) April temperature, (3) NAO_W_, and (4) ice concentration. We tested for possible confounding between these potential predictors, but as we found no worrying relationships between these variables we did not need to exclude any of them in any further analyses (Data S3). As we wanted to provide statistical control for population density, we also tested for the potential effect of population density with a lag of 1 year, that is, log_e_(*D*_*t*−1_)^1^. All variables, except lagged population density which was centered (i.e., subtracting the average from each observation), were detrended (Data S2). In order to further assess potential problems related to confounding and to test for any evidence of nonlinearity, we fitted a GAM using log_e_(*D*_*t*_) as the response where we modeled the smoothen effect of each key predictor separately (Data S4). As no key predictor showed any evidence for nonlinear relationship with log_e_(*D*_*t*_) we proceeded with analysis using linear models fitted using the lm function in the library stats (R Development Core Team 2011) and the gls function in the library nlme (Pinheiro et al. 2012) for R (see also Pinheiro and Bates 2000; Zuur et al. 2009). Model selection was done using the Akaike's Information Criterion (AIC) (e.g., Buckland et al. 1997; Anderson et al. 2000; Burnham and Anderson 2002; see Zuur et al. 2009:61 for an example using GAM). Specifically, we defined a set of different candidate models where we rescaled and ranked models relative to the model with the lowest AIC value (Δ_*i*_ denotes this difference for model *i*). Then, we selected the simplest model with a Δ _*i*_≤2. Model selection was performed in two steps (following Zuur et al. 2009: ch. 6): (1) as our data consist of regularly spaced time series, we assessed if a regular linear model violated the assumption of independence (i.e., if the residuals were temporally correlated), and (2) we kept the effect of all the key predictors but tested if the two-way interactions involving predation manipulation and population should be excluded/included in the final model used for inference (see Data S5 for technical details).

## Results

### Analysis 1 – temporal trends in population density

**Figure 3:**
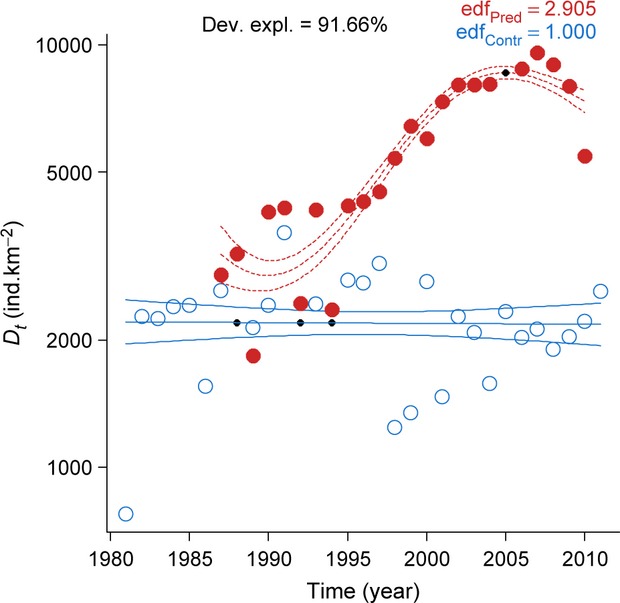
Temporal trends in population density (*D*_*t*_) for the control (open points and solid blue lines) and predator removal areas (closed points and dotted red lines; i.e., the area in which predators were actively removed). Predicted relationships (±1 SE) are from the GAMs presented in Table S1.1, whereas the points show the empirical data (closed black dots show the four imputed values for *D*_*t*_). Population density increased during the time period 1990–2005 in the predator removal population, whereas no trend in population density was apparent for the control population.

The GAM analysis of population density in relation to predator removal/control and year, including the predator removal/control × year interaction, explained a large proportion of the deviance in data (∼92%). The results showed that population density increased during the time period 1990–2005 in the predator removal population (Intercept = 7.693 [*P* < 0.001]; edf_treatment_ = 2.905 [*P* < 0.001]), whereas no trend in population density was apparent for the control population (Manipulation [predator removal] = 0.879 [*P* < 0.001]; edf_control_ = 1.000 [*P* = 0.947]) (Fig. 3; Data S1: Table S1.1A). In the treated population, the maximum range in predicted densities varied from ∼2850 (in the year 1990) to ∼8600 (2005) nest km^−2^, whereas the range in actual density was even higher as it ranged from ∼1850 (1989) to ∼9600 (2007) nests km^−2^ (Fig. 3). In the control area, however, the range in actual density varied from ∼770 (1981) to ∼3600 (1991) nests km^−2^ (Fig. 3).

### Analysis 2 – population dynamics: the Ricker model

**Figure 4:**
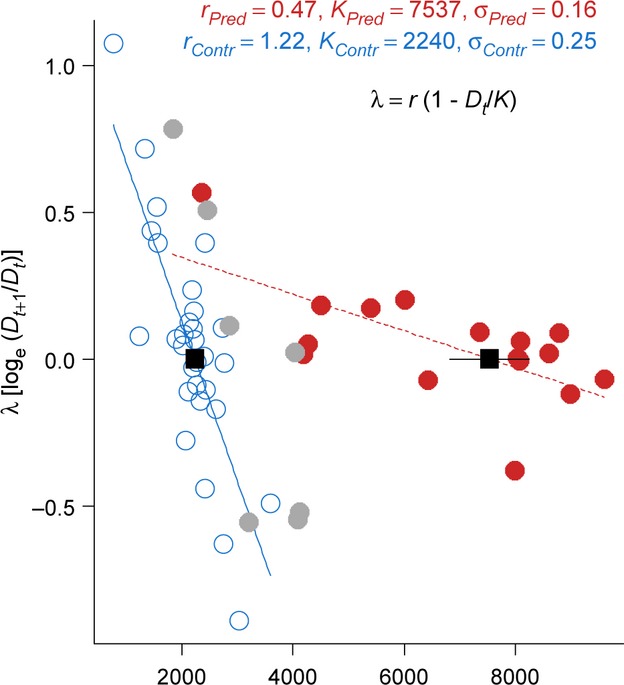
Population growth rate (λ) as a function of current population density (*D*_*t*_) for the control (open points and solid blue lines) and predator removal populations (closed points and dotted red lines). The lines show the predictions from the Ricker models fitted to the empirical data (see Table 1 for technical details regarding model parameters). Closed gray points shows the excluded data from before 1994 for the predator removal population (see Data S6 for an analysis where these data were included as well).

The results from the Ricker models revealed that the estimated carrying capacity (*K*) was 2240 (standard error [SE] = 89) nests km^−2^ for the control population and 7550 (SE = 710) nests km^−2^ for the predator removal population, whereas the control population experienced a higher intrinsic growth rate (*r*) compared to the predator removal population (Table 1). If we look at the relative difference between the populations these differences are even more striking: the difference in *r* was > twofold (control > predator removal), whereas the difference in *K* was approx. 3.5-fold (predator removal > control). Consequently, the estimated carrying capacity was approx. 5300 nest km^−2^ higher in the predator removal compared to the control population. The combined effect of these contrasting parameter values means that the population dynamics differed between the two populations (Fig. 4), which is in accordance with the previous analysis showing that the predator removal population increased during a time period in which the control population showed no apparent temporal trend (Fig. 3).

**Table 1:** Results from the Ricker model where population growth rate, that is, the change in population density (*D*) from 1 year (*t*) to the next (*t* + 1) [λ = log_e_ (*D*_*t*+1_−*D*_*t*_)], was predicted as a function of current population density (*D*_*t*_) for the (A) control, and (B) predator removal population (see Fig. 4 for a visualization of the model and the data)

Parameter	Estimate	St. err.	*t*	*P*
(A) Control population				
*r*	1.218	0.187	6.506	<0.001
*K*	2240.302	89.416	25.055	<0.001
(residual st. err. = 0.251, df = 25)				
(B) Predator removal population				
*r*	0.472	0.138	3.410	<0.001
*K*	7537.190	709.317	10.630	<0.001
(residual st. err. = 0.158, df = 13)				

*r* and *K* represent the estimated intrinsic rate of increase and the carrying capacity for the population, whereas the residual standard error provides an estimate of the precision of the model (see main text for details). Data from before 1994 for the predator removal population were excluded as the predator removal effort was low (see Data S6 for an analysis where these data were included as well.)

### Analysis 3 – climatic effects

**Figure 5:**
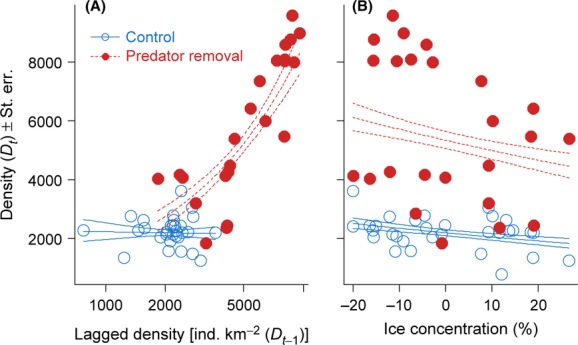
Population density (*D*_*t*_) as a function of (A) population density with a 1 year lag (*D*_*t*−1_) and (B) ice concentration. Each subplot shows data and predictions for the control (open points and solid blue lines) and the predator removal population (closed points and dotted red lines). Lines show the predictions from the model in Table 2 (except that the effect of log_e_(*D*_*t*−1_) was not centered in the figure) keeping all the other predictors at their average values. The curvature, which is especially visible for the predator removal population in the first subplot, is due to the fact that we have transformed our estimates (presented in Table 2) from log_e_ to natural scale. There was no significant relationship between lagged and current density for the control population, and this relationship was positive for the predator removal population (A). Ice concentration showed a significant negative relationship with population density in both populations (B).

A regular linear model was selected over the models containing any temporal correlation structures (where we considered autoregressive models of order 1 and 2; see Data S5). We selected the model with the lowest AIC value (where the closest model had a Δ_*i*_ of 4.5) from a set of a priori defined set of models (Data S5: Table S5.1). Consequently, the selected model had considerable more support in the data compared to the second ranked model. This model revealed that the main effect of treatment in the form of predation removal was as expected positive. More importantly, the positive interaction between lagged density and predator removal in combination with the lack of any main effect of lagged density (Table 2) revealed that (1) there was no significant relationship between lagged and current density for the control population, while (2) this relationship was positive for the predator removal population (Fig. 5A). Additionally, ice concentration showed a significant negative relationship with population density (Table 2), both in the control and the predator removal population (Table 2; Fig. 4). The other climatic variables, that is, April temperature, NAO_W_, or lagged July temperature, did not show any statistically significant relationships with population density (Table 2).

**Table 2:** Estimates from a linear model (*lm*) relating population density [log_e_(*D*_*t*_)] to treatment (i.e., a two-level factor: control and predator removal), lagged July temperature, Winter North Atlantic Oscillating Index (NAO_w_), ice concentration, April temperatures, and population density with a 1-year lag [log_e_(*D*_*t*−1_)]

Parameter	Estimate	St. err.	*t*	*P*
Population density: [log_e_(*D*_*t*_)]	*R*^2^ = 0.82			
Intercept	7.635	0.093	82.483	<0.001
Manipulation (predator removal)	0.580	0.125	4.630	<0.001
Lagged July temperature	0.066	0.048	1.360	0.181
NAO_w_	−0.015	0.019	−0.773	0.444
Ice concentration	−0.08	0.003	−2.573	0.014
April temperature	−0.012	0.011	−1.118	0.270
log_e_(*D*_*t*−l_)	−0.102	0.184	−0.554	0.583
(Manipulation)× log_e_(*D*_*t*−1_)	0.792	0.218	3.633	0.001
(*F =* 25.41; df = 7.41; *P <* 0.01)				

The intercept shows the average density for the control population, whereas the predator removal represents the difference between the averages for the control and predator removal populations (keeping all the other predictors at a constant of zero). Similarly, log_e_(*D*_*t*−1_) represents the estimated effect of previous population density for the control population, whereas the interaction between predator removal and log_e_(*D*_*t*−1_) represents the difference in effects comparing the predator removal and the control population (see main text for details).

## Discussion

This study demonstrates the negative effects of nest predators on population growth in common eiders. Actively limiting predator activity led to an increase in the carrying capacity with a factor of 3–4 compared to that found in the control population. Our results are thus in line with results from other predator removal studies (see Côté and Sutherland 1997 for a review).

The purpose of predator removal in an eider colony is to limit losses to egg predators (Stien et al. 2010). For a trapper/down harvester, this means more and better down due to reduced nest losses and fewer smashed eggs littering the down. Reduced egg depredation will lead to more young produced. Female eiders have strong site-specific site fidelity, almost always returning to their hatching colony when they start breeding at 2–3 years of age (Baillie and Milne 1982; Jónsson and Lúðvíksson 2013). In addition, females which experience good breeding conditions, that is, low egg predation, have a higher probability to nest within the same area next year (Bustnes and Erikstad 1993; Hanssen and Erikstad 2013). Reduced egg depredation will thus have both direct and indirect positive effects on eider populations: (1) directly as reduced predation rates increases hatch rates and hence the number of ducklings returning to breed themselves after 2–3 years, and (2) indirectly as females experiencing lowered predation are more inclined to breed in the same area in coming years (Hanssen and Erikstad 2013). This may also increase the probability of immigration, since the colony becomes a refuge with reduced predation pressure that could be attractive to individuals from surrounding areas. Also, improved breeding conditions may increase the proportion of adult females breeding in a given year (D'Alba et al. 2010). In addition, eider predation rates seem to decline when breeding densities increase (Ahlén and Andersson 1970; Mehlum 1991b). The result from this study, which demonstrates a rapid population growth when predation pressure is lowered, is likely to be an effect of both these processes.

Our study also shows that climate impacts the density of breeding populations as there was a negative relationship between April ice concentration and nest density. The Arctic climate has changed over the past 30 years. On the west coast of Spitsbergen, these changes include a reduction in sea ice and higher sea and air temperatures (e.g., Moe et al. 2009). This is in line with the trend for higher temperatures and reduced area and duration of ice and snow in the Arctic (IPCC 2007; AMAP 2011). In Greenland, for example, the date of snow melt has advanced 15 days during the period 1996–2005 (Høye et al. 2007), and the Arctic Ocean ice cover has been reduced by between 3% and 9% per decade (Serreze et al. 2007). For eiders and other ground nesting species, these changes are likely to have substantial effects as ice breakup around the breeding colonies prohibits predation from Arctic foxes (Mehlum 1991a; Svendsen et al. 2002; Chaulk and Mahoney 2012). In years with little ice and/or early ice break-up in the spring, the eiders also have earlier access to the benthic invertebrates that they rely upon for building up their energy reserves prior to breeding. In many long-lived birds, a part of the adult population may not breed in years of poor environmental conditions (Chastel et al. 1993; Cam et al. 1998). For eiders, it has been suggested that more “low-quality females” choose to nest in favorable years (Love et al. 2010). We show that a warmer climate will have positive effects on population density. This is supported by similar findings from Iceland and Canada (Jónsson et al. 2009; D'Alba et al. 2010). In addition, other studies have documented positive effects of earlier spring on timing of breeding (Love et al. 2010; Chaulk and Mahoney 2012), litter size (Lehikoinen et al. 2006; Chaulk and Mahoney 2012), body condition (Lehikoinen et al. 2006; Descamps et al. 2010), incubation costs and release of pollutants during incubation (Bustnes et al. 2012), and breeding success (Lehikoinen et al. 2006).

However, many natural eider populations seem to be showing a showing a negative trend in density or number, despite the fact that the climate is getting warmer (e.g., Gilchrist and Robertson 1998; Suydam et al. 2000; Hario and Selin 2002; Merkel 2004; Hario and Rintala 2006; but see D'Alba et al. 2010; Merkel 2010). This eider-climate-change paradox must mean that there are other factors limiting the positive effect of climate on reproduction. Increased nest predation by polar bears as a consequence of less ice and poor conditions for catching seal may be such an effect. In addition, a warmer climate may cause increased outbreak of infectious diseases (Descamps et al. 2011), indicating that population density might interact with climatic conditions in such a way that populations show a higher climatic vulnerability at high compared to low density (which has been shown for other long-lived species like e.g., reindeer: Bårdsen et al. 2011; Bårdsen and Tveraa 2012). It may also cause changes in the food chain and create less favorable feeding conditions (Waldeck and Larsson 2013). In the future, these negative climate effects may increase as sea acidification may affect calcifying organisms like shellfish heavily (Kleypas et al. 2006). Warmer climate may also facilitate the immigration of new species. The numbers of great skua *Stercorarius skua* show increasing trends on Svalbard, and this is a species affecting adult mortality of eiders negatively (B. Moe and S. A. Hanssen, pers. obs.).

In this semiexperimental study, we show that releasing the pressure from nest predators by human intervention lead to a rapid population increase toward a higher carrying capacity. We also show that climate affects population numbers as earlier ice-free areas lead to higher breeding population. Our ability to estimate *K*, which is a parameter strongly related to population regulation, using the Ricker model gives us valuable hints toward why we observe such contrasting temporal trends in density: (1) the control population were close to its carrying capacity (i.e., it were regulated) even when the sampling started, while (2) treatment in the form of predator removal seems to have increased the carrying capacity to such an extent that no effective regulation occurred until the end of the last millennium.

## Footnotes

1 This is approach is comparable to the use of population growth rate except that instead of predicting population change 1 year into the future (*t* + 1) current population density was predicted based on density 1 year back in time (*t*−1).

## >Supporting Information

**Data S1.** Imputation of data on population density and growth rate.

**Data S2.** Detrending continuous predictors.

**Data S3.** Assessing possible confounding.

**Data S4.** Univariate statistical approach.

**Data S5.** Model selection and the set of candidate models.

**Data S6.** The Ricker model fitted to the whole time series for the treated population.
