# Supplementary material for: A natural antipredation experiment: predator control and reduced sea ice increases colony size in a long-lived duck
Source: Ecol Evol. 2013 Sep 1;3(10):3554–64. doi: 10.1002/ece3.735 (PMC3797499; doi:10.1002/ece3.735)
Supplement: Supplementary file 1 [file ece30003-3554-SD1.docx]

SUPPLEMENT S1: IMPUTATION OF DATA ON POPULATION DENSITY AND GROWTH RATE

Four entries were missing during the course of the study: 3 values (1992, 1998 and 2004) for the control group and 1 (2005) value for the predator removal group. Generalized additive model (GAM) with a Gaussian family and a log-link function, which were defined using thin plate regression splines, a gamma value of 1.4 and k of 4 (see main text for details), was used to model temporal and spatial trends in population abundance and growth rate (Table S1.1). A model where the interaction between time and treatment group, i.e. where we modelled temporal relationships differently across areas, explained >92% of the variance in population density (Table S1.1a; Figure S1.1a). Consequently, we used predicted values from this model for the missing years for all analyses where density was included as either as a predictor or as a response variable.

Population growth rates (*λ*), which are deduced from population density [*λ* = log_e_(*D*_t+1_/*D*_t_)], were thus based on the full data set (i.e. it was based on the four imputed valued for density). Population growth rates showed, however, no temporal or spatial trends (Table S1.1b; Figure S1.1b). This means that the *λ* fulfils the stationary assumption, which is one important underlying assumption for most time series analysis, which implies that “(…) the probability laws that govern the behavior of the process do not change over time” (also see e.g. Shumway and Stoffer 2006, Cryer and Chan 2008:16).

# REFERNCES

Ims, R. A., Yoccoz, N. G., Bråthen, K. A., Fauchald, P., Tveraa, T. & Hausner, V. (2007) Can reindeer overabundance cause a trophic cascade? *Ecosystems,* **10,** 607-622.

Cryer, J. D. and K.-S. Chan. 2008. Time series analysis - with applications in R. Springer, New York, United States of America.

Shumway, R. H. and D. S. Stoffer. 2006. Time series analysis and its applications - with R examples. Springer, New York, United States of America.

Table S1.1. Generalized additive models (GAM) showing (a) population density (*D*_t_) and (b) growth rate (λ) was related to year and treatment (control and predator removal as the treatment groups). Estimated degrees of freedom (edf) provide an estimate of the degree of complexity in the relationship. As the deviance explained by the model (Dev. expl.) where density was the response was high (~92%) we used this model as a basis for imputing the four missing values needed in order to achieve complete time series for each treatment group (see Figure S1.1 for details).

Figure S1.1. Temporal trends in (a) population density (*D_t_*) and (b) population growth rates (*λ*) for the control (open points and solid blue lines) and predation removal areas (closed points and dotted red lines; i.e. the area in which predators were actively removed). Predicted relationships (± 1 SE) are from the GAMs presented in Table S1.1, whereas the points show the empirical data (closed black dots show the four imputed values for *D_t_*).
