# Supplementary material for: A natural antipredation experiment: predator control and reduced sea ice increases colony size in a long-lived duck
Source: Ecol Evol. 2013 Sep 1;3(10):3554–64. doi: 10.1002/ece3.735 (PMC3797499; doi:10.1002/ece3.735)
Supplement: Supplementary file 2 [file ece30003-3554-SD2.docx]

SUPPLEMENT S2: DE-TRENDING CONTINUOUS PREDICTORS

All continuous variables except population density were de-trended using a model that accounted for the effect of year. We used the residuals from a generalized additive model (GAM) with a Gaussian family and a log-link function, which were defined using thin plate regression splines, a gamma value of 1.4 and k of 4 (see main text for details) for one predictor variable at a time (figure S2.1).

Figure S2.1. Temporal trends for (a) Lagged (2 year) July temperature (July temp_lag2, °C), (b) North Atlantic Oscillation winter index (NAO_W_), (c) Ice concentration (%), and (d) April temperature (April temp, °C). Predicted relationships (± 1 SE) are from the GAMs.
