# Supplementary material for: A natural antipredation experiment: predator control and reduced sea ice increases colony size in a long-lived duck
Source: Ecol Evol. 2013 Sep 1;3(10):3554–64. doi: 10.1002/ece3.735 (PMC3797499; doi:10.1002/ece3.735)
Supplement: Supplementary file 3 [file ece30003-3554-SD3.docx]

SUPPLEMENT S3: ASSESSING POSSIBLE CONFOUNDING

Figure S3.1. Person’s product moment correlation coefficients assessing the relationship between possible predictors (see Supplement S2 for details on the variable notations used).
