# Supplementary material for: A natural antipredation experiment: predator control and reduced sea ice increases colony size in a long-lived duck
Source: Ecol Evol. 2013 Sep 1;3(10):3554–64. doi: 10.1002/ece3.735 (PMC3797499; doi:10.1002/ece3.735)
Supplement: Supplementary file 4 [file ece30003-3554-SD4.docx]

SUPPLEMENT S4: UNIVARIATE STATISTICAL APPROACH

In order to test the effect of each (de-trended) climatic predictors separately, we fitted models using density (*D*_t_) as the response, and as we expected previous population density to be important we also kept previous density [log_e_(*D*_t_); the effect of this variable, which was not de-trended, was assumed linear] as a covariate in these analyses. More specifically, we used generalized additive models (GAM) with a Gaussian family and a log-link function, which were defined using thin plate regression splines, a gamma value of 1.4 and a k of 4 (see main text for details: Table S4.1; Figure S4.1). We also kept the interaction between the climatic predictor and manipulation group, which means that we modelled the smoothened effect of each climatic variable differently across populations. All these models provided good fits to the data as they all explained 74-78% of the deviance (Table S1.1a; Figure S1.1a).

Table S4.1 Generalized additive models (GAM) showing how population density (*D*_t_) was related to treatment (control and predator removal as the treatment group) and previous density [log_e_(*D*_t_)]. Estimated degrees of freedom (edf) provide an estimate of the degree of complexity in the relationship.


Figure S4.1. Population density (*D*_t_) as a function of (a) lagged (2 year) July temperature, (b) winter North Atlantic Oscillation (NAOw), (c) ice distribution, and (d) April temperatures. Predicted relationships (± 1 SE) are from GAMs where we account for the effect of population density with a one year lag (i.e. *D*_t-1_; see Table S4.1 for details).
