# Supplementary material for: A natural antipredation experiment: predator control and reduced sea ice increases colony size in a long-lived duck
Source: Ecol Evol. 2013 Sep 1;3(10):3554–64. doi: 10.1002/ece3.735 (PMC3797499; doi:10.1002/ece3.735)
Supplement: Supplementary file 5 [file ece30003-3554-SD5.docx]

SUPPLEMENT S5: MODEL SELECTION AND THE SET OF CANDIDATE MODELS

Selecting the models used for inference was performed within a model selection framework using *Akaike’s Information Criterion* (AIC) (e.g. [Buckland et al. 1997](#_ENREF_3), [Anderson et al. 2000](#_ENREF_1), [Burnham and Anderson 2002](#_ENREF_4)) as follows:

1. We formed a set of candidate models where models were rescaled and ranked relative to the model with the lowest AIC value (Δ*_i_* denotes this difference for model *i*).
2. We selected the simplest model, i.e. the model with the fewest degrees of freedom, with a Δ*_i_* ≤2. In all the analyses we kept the key predictors in the models based on our *a priori* expectations, whereas covariates and the interactions was excluded and included in the model used for inference based on how they affected the AIC (and the Δ*_i_*).

As we were working on time series data it was natural to assess the extent in which the residuals were autocorrelated. This was graphically assessed by fitting a regular linear model (fitted using the gls function in the nlme library), and then by applying regular autocorrelation functions (using the acf function) on the residuals (for each population separately). This approach indicated that no apparent autocorrelation in the residuals (Figure S5.1). Then we re-fitted this model with two different correlation structures; one assuming a autoregressive order of 1 and 2 (see [Pinheiro and Bates 2000](#_ENREF_5), [Zuur et al. 2009 for details](#_ENREF_6)), but as these models did not provide better fits compared to the regular linear model (Table S5.1, see also Figure S3.1) we used the regular model in the further data analyses (i.e. selecting between models containing different predictors and our use of a final model for inference (see main text for details).

# REFERENCES

Anderson, D. R., K. P. Burnham, and W. L. Thompson. 2000. Null hypothesis testing: problems, prevalence, and an alternative. Journal of Wildlife Management **64**:912-923.

Bolker, B. M., M. E. Brooks, C. J. Clark, S. W. Geange, J. R. Poulsen, M. H. H. Stevens, and J. S. S. White. 2009. Generalized linear mixed models: a practical guide for ecology and evolution. Trends in Ecology & Evolution **24**:127-135.

Buckland, S. T., K. P. Burnham, and N. H. Augustin. 1997. Model selection: an integral part of inference. Biometrics **53**:603-618.

Burnham, K. P. and D. R. Anderson. 2002. Model selection and multimodel inference: a practical information-theoretic approach. Second edition. Springer, Inc., New York, USA.

Pinheiro, J. C. and D. M. Bates. 2000. Mixed effect models in S and S-PLUS. Springer, New York, USA.

Zuur, A. F., E. N. Ieno, N. J. Walker, A. Saveliev, A., and G. M. Smith. 2009. Mixed effects models and extensions in ecology with R. Springer, New York, USA.

Figure S5.1 Plotting the autocorrelations for the normalized residuals (separated by population) from model 1 in Table S5.1.Table S5.1. The relative evidence for each candidate model (i) based on differences in AIC values (Δ*_i_*). The model in underlined was selected and used for inference, whereas the predictors in **bold** was kept in all models. All models contain the same predictors as model 16 in Table S5.2.

^a^Models were fitted using the gls function in *R*.

^b^Models were fitted using the gls function in *R*, with the following arguments: *correlation = corARMA(p = x, q = 0, form = ~1|Manipulation)*, where x was set to 1 and 2 for the AR-1 and AR-2 model respectively.

^c^*K* denotes the number of parameters, whereas the number of observations (*n*) was 49.

^d^Restricted maximum likelihood (REML) fitted models were used when these models were compared ([Pinheiro and Bates 2000](#_ENREF_5), [Zuur et al. 2009](#_ENREF_6)).

Table S5.2. The relative evidence for each candidate model (i) based on differences in AIC values (Δ*_i_*). The model in underlined was selected and used for inference, whereas the predictors in **bold** was kept in all models.

^a^The predictor in **bold** was kept in all models based on our *a priori* expectations.

^b^*K* denotes the number of parameters, whereas the number of observations (*n*) was 49.

^c^Maximum likelihood (ML) fitted models were used when these models were compared ([Pinheiro and Bates 2000](#_ENREF_5), [Zuur et al. 2009](#_ENREF_6)).
