# Supplementary material for: A natural antipredation experiment: predator control and reduced sea ice increases colony size in a long-lived duck
Source: Ecol Evol. 2013 Sep 1;3(10):3554–64. doi: 10.1002/ece3.735 (PMC3797499; doi:10.1002/ece3.735)
Supplement: Supplementary file 6 [file ece30003-3554-SD6.docx]

SUPPLEMENT S6: THE RICKER MODEL FITTED TO THE WHOLE TIME-SERIES FOR THE TREATED POPULATION

Table 1. Results from the Ricker-model where population growth rate, i.e. the change in population density (*D*) from one year (*t*) to the next (*t*+1) [λ = log_e_ (*D*_t+1_ - *D*_t_)], was predicted as a function of current population density (*D*_t_) for the: (a) control; and (b) predator removal population (see Fig. 2 for a visualization of the model and the data). *r* and *K* represents the estimated intrinsic rate of increase and the carrying capacity for the population, whereas the residual standard error provides an estimate of the precision of the model (see main text for details).


Fig. S6.1. Population growth rate (λ) as a function of current population density (*D*_t_) for the (a) control (open points and solid blue lines) and (b) predator removal populations (closed points and dotted red lines). The lines show the predictions from the Ricker models fitted to the empirical data (see Table 1 for technical details regarding model parameters).
